# Subjective Evaluation of Performance in a Collaborative Task Is Better Predicted From Autonomic Response Than From True Achievements

**DOI:** 10.3389/fnhum.2020.00234

**Published:** 2020-07-17

**Authors:** Alexander Maye, Jürgen Lorenz, Mircea Stoica, Andreas K. Engel

**Affiliations:** ^1^Department of Neurophysiology and Pathophysiology, University Medical Center Hamburg-Eppendorf, Hamburg, Germany; ^2^Laboratory of Human Biology and Physiology, Faculty of Life Science, Applied Science University, Hamburg, Germany

**Keywords:** biophysical methods, self-perception, joint attention, embodied cognition, human behavior

## Abstract

Whereas the fundamental role of the body in social cognition seems to be generally accepted, elucidating the bodily mechanisms associated with non-verbal communication and cooperation between two or more persons is still a challenging endeavor. In this article we propose a fresh approach for investigating the function of the autonomic nervous system that is reflected in parameters of heart rate variability, respiration, and electrodermal activity in a social setting. We analyzed autonomic parameters of dyads solving a target-tracking task together with the partner or individually. A machine classifier was trained to predict the subjects' rating of performance and collaboration either from tracking error data or from the set of autonomic parameters. When subjects collaborated, this classifier could predict the subjective performance ratings better from the autonomic response than from the objective performance of the subjects. However, when they solved the task individually, predictability from autonomic parameters dropped to the level of objective performance, indicating that subjects were more rational in rating their performance in this condition. Moreover, the model captured general knowledge about the population that allows it to predict the performance ratings of an unseen subject significantly better than chance. Our results suggest that, in particular in situations that require collaboration with others, evaluation of performance is shaped by the bodily processes that are quantified by autonomic parameters. Therefore, subjective performance assessments appear to be modulated not only by the output of a rational or discriminative system that tracks the objective performance but to a significant extent also by interoceptive processes.

## 1. Introduction

Today the idea that social cognition is not a purely mental phenomenon but also involves the body seems to be accepted by many researchers. Yet we need to better understand the bodily mechanisms during non-verbal social interaction. Relevant phenomena range from the tactile and proprioceptive perception of touches, forces, and torques that are produced by physical contact between persons either directly or mediated by objects, over observation or gestures involved in interacting across peripersonal space, to complex emotional processes that characterize shared experiences and behavior between individuals. To understand how individuals perceive other agents and control social behavior therefore requires the examination of the relationship between the autonomic nervous system and interoceptive and emotional functions.

In this respect the analysis of heart rate variability (HRV) as an autonomic indicator provides a particularly interesting approach. Normal heartbeat is automatically generated by autorhythmic cells in the sinoatrial node. The cardiac pacemaker possesses a substantial level of autonomy from the brain in that the basic activity pattern continues even when the heart is denervated. Still this activity is permanently modulated by the brainstem through the sympathetic and parasympathetic nervous system, accelerating and decelerating heart rate, respectively. We consider HRV as an index for the nervous and hormonal signals that distinctly modulate the strength of sympathetic and parasympathetic actions on the heart pacemaker, the sinoatrial node, which results in changes of inter-beat intervals (Shaffer and Ginsberg, 2017). Parasympathetic modulation of cardiac activity by the brain is mediated through the right vagus nerve; sympathetic modulation is exerted through inputs from postganglionic efferents originating in the stellate ganglion. The brain, in turn, receives afferent signals from aortic, carotide, and pulmonary baroceptors through the vagus nerve (Ellis and Thayer, 2010). Respiratory modulation of heart rate with increases during inspiration and decreases during expiration is called respiratory sinus arrhythmia (RSA, Berntson et al., 1993). Although RSA grossly reflects the rhythmical fluctuation of pulmonary vagal afferent and cardiac vagal efferent effects upon the sinoatrial node in synchrony with the breathing cycle, experimental and anatomical evidence indicate additional RSA-independent sources of cardiac vagal tone (Grossman and Taylor, 2007; Farmer et al., 2016). Together with electrodermal activity (EDA), which is typically considered as an index of sympathetic activity (Dawson et al., 2007), parameters of the respiration rhythm (RR) and HRV therefore may be considered as a complex status indicator of the autonomic nervous system.

A mutual brain-viscera interaction has been highlighted as early as in the nineteenth century (Charles et al., 1998) for the understanding of the interplay between emotion and cognition. Damasio (1999) considers visceral input as part and parcel of emotion and suggests that in particular background feelings “are a faithful index of momentary parameters of inner organism state” which have the “temporal and spatial shape of ... the striated muscle of heart and chest” as core ingredients (p. 286f.). The central autonomic network (Benarroch, 1993, 2014), linking the brainstem with forebrain structures through feed-back and feed-forward loops, is responsible for generating visceromotor, neuroendocrine, and behavioral responses that are flexibly adapted to environmental demands (Thayer and Lane, 2000). Indeed several studies have found a relation between HRV and the adaptive and functional top-down and bottom-up cognitive modulation of emotional stimuli (Park and Thayer, 2014). Since activity in anterior regions of the prefrontal cortex correlates with HRV specifically during emotionally challenging situations, individuals with high HRV may be particularly efficient in recruiting the “social cognition” network in emotional contexts (Beffara et al., 2016). Correlations of emotional state have been shown to exist with individual HRV parameters (Zhu et al., 2019), such as mean heart rate (Yoshino et al., 2011; Choi et al., 2017) or the high-frequency component of heart rate fluctuations (Lane et al., 2009), as well as with subsets of HRV parameters (Rainville et al., 2006). Moreover, higher levels of heart rate synchrony have been suggested as a marker of interpersonal trust (Mitkidis et al., 2015). Several studies revealed correlations of the emotional state with parameters of RR (Del Negro et al., 2018) and EDA (Sequeira et al., 2009). Real-time feedback about HRV coherence in pairs or groups of people is used to investigate whether learning to regulate physiological coherence helps increasing social coherence, leading to increased prosocial behaviors, improved communication, cooperation, creativity, and decision making (McCraty, 2017).

The influence of visceral information on perceptual processes and cognitive functions however is less well-explored. Recent findings show that heartbeat-evoked neural activity can modulate perceptual thresholds and shape visual conscious experience (Park et al., 2014). This led to the hypothesis that the neural representation of visceral information, projected through multiple anatomical pathways to a network of brain regions including posterior insula, ventral anterior cingulate cortex, amygdala and somatosensory cortex, constitutes an implicit frame which could explain the subjective nature of perceptual experience and link it with emotions and the notion of the self (Park and Tallon-Baudry, 2014). This hypothesis gains support from the observation that heartbeat-evoked neural responses co-vary with the self-relatedness of ongoing spontaneous thoughts (Babo-Rebelo et al., 2016). Heart rate and EDA have been found to correlate with various dimensions of the subjective experience of playing a computer game, linking quantitative parameters with the quality of user experience (Drachen et al., 2010).

The study we present in this article is geared to contribute at least two novel aspects to this interesting line of research. The first is to go beyond establishing correlative relations and explore possibilities for actually predicting the outcome of the evaluation of the subjective experience. We approach this question by training a machine classifier to predict ratings of subjective experience from autonomic parameters and analyzing the prediction performance. If the trained model performs above chance level, we conclude that the autonomic response must be informative about the result of this assessment.

The second contribution of our study to the growing knowledge about brain-visceral interaction is a fresh approach for analyzing the relation between autonomic parameters and behavioral responses. The typical approach selects a single parameter or a few and analyzes how they change between normal vs. clinical conditions (Shaffer and Ginsberg, 2017). In contrast, we here follow a strategy that is inspired by machine learning approaches. Rather than considering HRV, RR, or EDA separately and analyzing individual parameters or small subsets thereof, we conceive of autonomic parameters as a feature vector which is characteristic for the stable dynamics of the body. One advantage of this approach is that it allows us to discover patterns in the parameter set which are more complex than increases or decreases of individual parameters.

We study the interaction between autonomic state and subjective experience during a joint target tracking task. Each partner controlled one of two perpendicular axes of motion, and together they had to roll a virtual ball as close as possible to a target that moved in an oblique direction. After each trial, a subjective assessment of their own performance, the partner's performance and the collaboration was requested from both participants. We investigate a potential relation between this subjective experience of performance and parameters of HRV, RR, and EDA, and we contrast it with the actual performance measured by the tracking error. We elucidate potential differences between a collaborative condition, in which subjects jointly controlled the ball, and a condition in which they solved the task individually. We do not consider the learning process for acquiring the skill to solve task here; therefore, subjects exercised the task for several days, and we analyzed the data after performance had stabilized.

Two non-exclusive hypotheses about the basis of the processes for the subjective evaluation of performance in the task will be investigated with our approach. Hypothesis 1 (H1) entails that the assessment of performance is driven by the subject's tracking of the task performance, as indexed by an objective criterion, i.e., by evaluating the tracking error. This hypothesis follows from the assumption that the individual utilizes the recall of memorized behavioral performance parameters to retrospectively rate performance. Support for H1 would be gained from good performance of the classifier for predicting ratings from the tracking error. The main idea of hypothesis 2 (H2) is that a feeling about the own performance, rather than objective discrimination, guides the ratings in the self-assessment. We postulate that this feeling about the own performance is reflected in the autonomic response and hence consider good performance of the classifier for predicting ratings from autonomic parameters as support for H2. H2 is closely related to the idea that visceral bodily states can influence how humans perceive their own actions, whereas H1 is more compatible with the view that feedback on overt motor behavior determines this experience.

## 2. Materials and Methods

### 2.1. Experimental Setup

The cooperative task, a dual target-tracking task, was implemented on a tablet computer (iPad2, Apple Inc.). By tilting the tablet, subjects had to move a virtual ball into the center of a moving circle. The target circle moved along a straight line at a fixed speed, but reversed its direction of movement at random intervals. The animation of the virtual ball followed the kinematic equations resulting from Newton's second law to make its behavior naturalistic.

**Figure 1:**
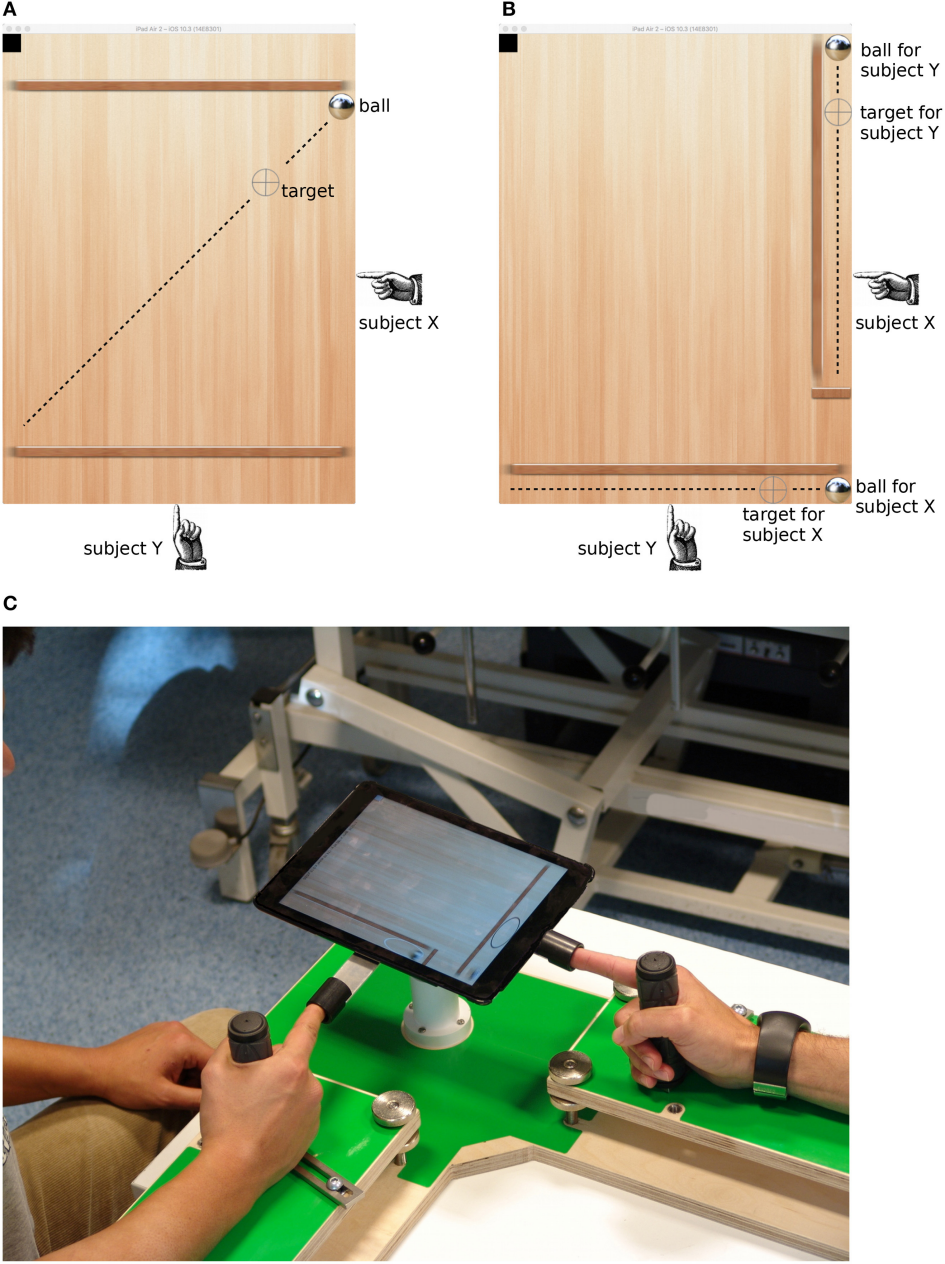
Screenshots from the tablet computer showing the collaborative **(A)** and individual **(B)** conditions. The gray crosshair is the target that moved at constant rate along the dashed line (the line only illustrates the motion path; it was not visible to the subjects). Subjects tilted the tablet to make the ball roll to the target location. The black square in the upper left corner was used to synchronize the tablet with the amplifier and was not visible to the subjects. **(C)** Picture of the experimental setup.

Each player controlled only one axis of the tablet. In the collaborative condition, a single target moved on a diagonal line, and subjects cooperatively moved the ball toward the target. In the individual condition, there were two confinements along the main axes of the tablet, each containing a ball and a target, and subjects tracked the target in their respective confinement independently from the partner. Figures 1A,B show screen shots of the two conditions.

In order to constrain movements of the subjects, we developed a custom-made frame consisting of two armrests arranged in an L-shape, handles on each armrest, and a ball joint support for the tablet computer at the intersection of the armrests (see Figure 1C). Subjects grasped the handle with their right hand and extended the index finger into a thimble which was attached at each side of the tablet computer. The ball joint held the tablet's balance and allowed the subjects to tilt it along the respective main axes by lifting or lowering the index finger with minimal physical effort. As the friction of the virtual ball was low, small finger movements were sufficient to move the ball around. Subjects did not report problems with controlling the ball or fatigue.

The experimental setup and the participants were placed in an electrically and acoustically shielded chamber. Subjects were instructed to not communicate verbally or gesticulate during the experiment, i.e., during the game or when submitting ratings. Compliance with this instruction was checked by the experimenter through a camera mounted in the recording chamber. Subjects were also instructed to not rotate the tablet around the vertical axis (by moving the index finger in a plane parallel to the table), but there was no mechanism for preventing such movements. Rotations in the horizontal plane, however, could not affect the ball's movement, and we have no indication that subjects used such movements as a means of covert communication.

### 2.2. Subjects and Study Protocol

Twenty eight subjects participated in the study (20 females, mean age 25.18 ± 3.86 years). They were right-handed and reported to be in healthy condition. Subjects gave written informed consent before commencing the experiment. As part of the procedure for obtaining informed consent from the participants, they were instructed not to smoke, consume drugs or drink alcohol or coffee before the experiment. Apart from contraceptives, participants were free from medication. The study was approved by the ethics committee of the medical association of the city of Hamburg. The experiments were performed in accordance with the Declaration of Helsinki.

Subjects were paired in 14 dyads. In all but two dyads who were exclusively female, teams were mixed-gender on at least one of the 2 days when data were recorded. On the first day of the experiment, all participants except 4 (2 dyads) declared to never have met the respective partner before.

Each dyad exercised the task on 6 consecutive days, because we aimed at analyzing the processes when task performance was dynamically stable. On each day, they completed 7 trials in either condition (collaborative/individual). The order of the conditions was randomized. Each trial lasted for 120 s. Immediately after a trial, the experimenter requested the subjects to rate their performance by asking them:

R1 : “Please rate your own performance.”
R2 : “Please rate your partner's performance.”
R3 : “Please rate the collaboration.”

Subjects made their assessment by selecting a number between 1 and 9 (1-worst performance, 9-best) on a small remote control which they held in their left hands underneath the armrest so that the partner could not see their selection. R2 and R3 were called out only after a collaborative trial. Whereas R1 and R2 measured the subjects' impression of their performance as individuals, R3 was targeted at their performance as a team. The ratings were designed to capture different aspects in the social interaction of the subjects on the task and to facilitate correlation analyses with signatures of their body and brain activity.

On days 7 and 8, they performed the same procedure, but in addition, electrophysiological data were recorded (see below). On day 7, subjects cooperated with the same partner like the 6 days before, whereas on day 8 they were paired with a different but equally trained subject. In order to improve statistical power, but also because we here are not concerned with the differences between collaboration with a known vs. new partner, we combined the data from days 7 and 8.

### 2.3. Recording Physiological Signals and Calculating Autonomic Parameters

We recorded EEG, EMG, ECG, RR, and EDA simultaneously from the two subjects in each dyad using an EEG amplifier (ActiveTwo AD-box, BioSemi instrumentation) and an amplifier for physiological signals (MP35, Biopac Systems Inc.). Both amplifiers were synchronized by a common clock. Here we analyze ECG, RR, and EDA data only; results of EEG and EMG data analyses will be reported elsewhere.

The air in the recording chamber was conditioned to have a temperature of 21°C and a humidity of 40%. The interior was illuminated by 4 × 25 W LED lights on the ceiling. Except for day 8, when participants interacted with a new partner, data were recorded at the same time of the day, which differed between the teams though.

Two ECG electrodes were placed below the upper medial clavicle and on the Erb point (Eindhoven 2). ECG was sampled at 2,048 Hz. Respiration was recorded by a strain sensor on an elastic belt which subjects wore around their chest. Electrodes for recording skin conductance (EDA) were placed on the distal phalanx of the index and middle fingers on the left hand. RR and EDA were sampled at 10 Hz and a resolution of 24 bit.

ECG data analysis started by detecting R-peaks using the *qrsdetect* function in the Biosig toolbox (Vidaurre et al., 2011) in Matlab (The Mathworks). Correctness of QRS detection was checked visually for each subject. Detection of the QRS-complex resulted in so-called normal-to-normal intervals (NN) from which then HRV parameters were calculated using the *heartratevariability* function of the toolbox. Frequency-domain parameters were calculated using autoregressive modeling. Using a fast Fourier transform did not qualitatively change the results.

We used a subset of the HRV parameters that are described in (Camm et al., 1996) and, additionally, Poincaré-map parameters (SD1, SD2, r-RR; Brennan et al., 2001). Table 1 lists all HRV parameters together with a short description of what they represent; a comprehensive explanation and their clinical relevance is given in Shaffer et al. (2014). It has to be pointed out that the HRV parameters are not independent measures of cardiac activity; rather, several of them are correlated to various degrees (Shaffer and Ginsberg, 2017).

**Table 1:** HRV parameters.

**HRV parameter**	**Description**
ecg_mean	Mean duration of NN intervals
ecg_SDNN	Standard deviation of the NN interval
ecg_RMSSD	Square root of the mean of the squared differences between successive NNs
ecg_NN50, ecg_pNN50	Number of pairs of successive NNs that differ by more than 50 ms (NN50count) and its ratio to the total number of intervals (pNN50)
ecg_SD1, ecg_SD2	Width and length of the Poincaré plot
ecg_r_RR	Correlation coefficient in the Poincaré plot
ecg_VLF, ecg_LF, ecg_HF, ecg_tot_pwr	Power in three frequency bands (0.009–0.04, 0.04–0.15, 0.15–0.4 Hz) and total power
ecg_LF/HF	ratio of LF to HF power
ecg_LFnu, ecg_HFnu	LF and HF in normalized units, i.e., the relative value in proportion to the TotalPower minus VLF

In women, HRV parameters are known to be modulated by the phase of the menstrual cycle (Sato et al., 1995; Yildirir et al., 2001; Brar et al., 2015). There are a number of other factors, however, which also affect HRV. Age and body-mass index, for example, have been shown to exert a stronger modulation than menstrual cycle (Vallejo et al., 2005; Zhang, 2007). Likewise differences in HRV between female and male participants in our cohort had to be expected (Zhang, 2007). Since here we are not interested in the distribution of individual HRV parameters across the population, but rather in the predictive information when considered jointly, we consider menstrual cycle as one of many factors that give rise to inter-individual differences of HRV parameters and devise our methods to cope with these differences.

The respiration signal was band-pass filtered between 0.05 and 0.5 Hz, and the instantaneous breathing rate was determined from the zero-crossings of the resulting signal. Interval durations shorter than 0.5 s were considered as artifacts and removed. From the Fourier power spectrum of the filtered signal, the integrals in the frequency bands from 0.07 to 0.14 and 0.15 to 0.5 Hz, normalized by the total power, yielded spectral power features in the mid and high frequency bands, respectively (Hidalgo-Muñoz et al., 2018). RR parameters are listed in Table 2.

**Table 2:** RR parameters.

**RR parameter**	**Description**
resp_mean	Mean breathing rate
resp_std	Standard deviation of the breathing rate
resp_MF	Spectral power in the middle frequency band (0.07–0.14 Hz)
resp_HF	Spectral power in the high frequency band (0.15–0.5 Hz)

Skin conductance was decomposed into a tonic skin conductance level (SCL) and a transient skin conductance response (SCR) (Boucsein, 2010) using the continuous decomposition analysis implemented in the LEDALAB toolbox (Benedek and Kaernbach, 2010). Since SCR events may reflect stimulus-related as well as non-specific responses, and to avoid the intricacies involved in finding thresholds which define such events, we followed the approach suggested in Zhang et al. (2018) and considered the integrated SCR (iSCR) that was calculated by integrating the SCR time courses across 10 s non-overlapping time windows. SCL was treated in the same way. The means and standard deviations across the trial yielded the EDA parameters listed in Table 3.

**Table 3:** EDA parameters.

**EDA parameter**	**Description**
iscl_mean	Mean of the integrated SCL (integration across 10 s non-overlapping time windows)
iscl_std	Standard deviation of the integrated SCL
iscr_mean	Mean of the integrated SCR (integration across 10 s non-overlapping time windows)
iscr_std	Standard deviation of the integrated SCR

For the statistical analysis of correlations between ratings and autonomic parameters, we assumed a significance threshold of 0.05 and used the false discovery rate (FDR, Benjamini and Hochberg, 1995) to correct for multiple comparisons.

### 2.4. Classification

We employed quadratic discriminant analysis (QDA) as a model for the relation between autonomic parameters or objective performance and ratings. QDA is a variant of linear discriminant analysis (LDA Rao, 1948; Hastie et al., 2001) which allows for different covariance matrices for each class and hence more complex decision boundaries. Accuracies from a linear classifier were always lower, which relates to the finding that the inclusion of quadratic terms seemed to improve model accuracy (Beffara et al., 2016). Although the problem naturally is one of ordinal classification or regression, none of the corresponding methods that we tested (LASSO, random forests, support vector machines) outperformed QDA. We used the implementations of QDA and LDA that are provided in Matlab (*classify*) and custom scripts.

Models were trained on pooled data from all subjects (*N* = 28subjects × 14trials × 2days = 784samples). In order to combine autonomic data from all subjects, we normalized data by linearly mapping them to the interval [0, 1]. We also tried z-scores for normalizing the distribution of each parameter per subject to have zero mean and unit variance, but this did not qualitatively affect the results.

**Figure 2:**
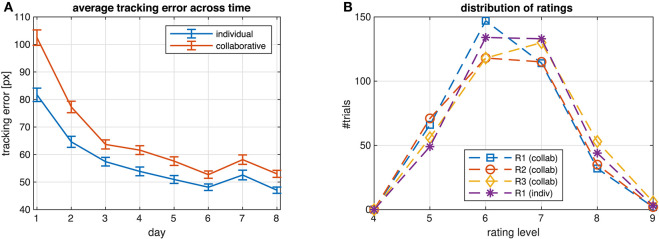
**(A)** Average tracking error across all subjects for each day of participating in the study. ECG was recorded on days 7 and 8, RR and EDA on every day. **(B)** Distribution of ratings R1-3 from all subjects in the two conditions (collaborative/individual) on days 7 and 8 after correcting outliers and eliminating rating levels with an insufficient number of samples.

Ratings were not normalized, but we checked for outliers of the average ratings per subject with respect to the whole population. If the median of the ratings from a subject was more than 1.5 interquartile ranges above the upper quartile or below the lower quartile, we corrected ratings from this subject by subtracting the difference between the subject's median rating and the median of all other subjects' median ratings. Ratings of two subjects were corrected in this way. In order to equalize the number rating levels across the three ratings R1-3 and two conditions (collaborative/individual), and to eliminate rating levels with an insufficient number of samples for classification, we used only those trials where the rating was among the six most frequent levels. This resulted in discarding 2/6/5 trials from R1/2/3 in the collaborative condition and 3 trials from R1 in the individual condition. The resulting frequencies of the *L* = 6 rating levels are shown in Figure 2B.

Objective task performance was measured by calculating the tracking error. To evaluate the performance as a team, the cumulative Euclidean distance (in pixels) between the target and the ball across the duration of the trial was calculated. The individual objective performances were given by the cumulative distance (in pixels) in x-/y-direction between the target and the ball across the trial duration.

Two sets of classifiers were trained to predict ratings. One set was trained on the tracking errors. For each rating category R1–3, the corresponding tracking error was used as a measure of the subject's or the dyad's objective performance: Ratings of own performance (R1) were predicted from the tracking error along the axis that the subject controlled; ratings of the partner's performance (R2) were predicted from the tracking error along the axis that the partner controlled; and ratings of collaboration (R3) were predicted from the Euclidean distance between the ball and the target. The other set of classifiers was trained on the autonomic parameters.

Model performance was calculated by leave-one-sample-out cross-validation: One sample was selected from the data set, and the classifier was trained on the remaining samples. The trained classifier was then used to predict the rating of the selected sample, and the output was compared with the observed ratings. Repetition of this procedure for each sample in the data set yielded an estimate of the model's performance. To corroborate the results, and to investigate how the model responds to data from an unseen subject, we also employed leave-one-subject-out cross-validation: Here the classifier was trained on data from all but one subjects and tested on data from this subject. As this classifier captures common properties across the cohort of participants, we call this classifier the population model.

In order to reduce the redundancy between the numerous autonomic parameters and thereby improve classification performance, we employed backward feature selection for each of the three ratings and two conditions. Successively, each parameter was temporarily omitted from the data set and the resulting classification performance was determined using leave-one-sample-out cross-validation. The parameter that, when omitted from the data set, yielded the strongest performance increase was then permanently removed, and the procedure was repeated until no further performance improvements were achieved. The reduced set of parameters was then used to determine the final model performance.

Importance of the remaining parameters was estimated by permuting them individually and gauging the decrease in model performance. Parameters that cause large decreases under permutation can be considered more important than those with smaller decreases (Breiman, 2001). A data set with a permuted parameter was generated by replacing in each of the *N* samples of the original data set the value of the respective parameter with the values in all other samples, yielding a new data set of size *N*(*N* − 1). Model performance on this data set was then evaluated using leave-one-sample-out cross-validation, and the difference to the model performance on the original data set was taken as a measure of parameter importance.

Since the number of samples for each rating level was far from equal (see Figure 2B), we report model performance in terms of F1-scores rather than prediction accuracies. The F1-score is the harmonic mean of *recall* and *precision* of a classifier,

$$F_{1}=\frac{2}{\frac{1}{recall}+\frac{1}{precision}},$$

whereby

$$recall=\frac{1}{L}\overset{L}{\underset{l=1}{\sum }}\frac{TP_{l}}{P_{l}}\;\text{and}\;precision=\frac{1}{L}\overset{L}{\underset{l=1}{\sum }}\frac{TP_{l}}{TP_{l}+FP_{l}}$$

are the averages of the class-specific recall and precision which are calculated from the number of true positive (TP), total positive (P), and false positive (FP) classifications.

The cross-validation methods yielded a single F1-score per condition and rating. We assessed the likelihood of obtaining the reported F1-scores by chance by running permutation tests on all classifiers (Good, 2013). In each of the 1,000 repetitions, we trained and tested the classifier on a data set in which the structure had been destroyed by randomly permuting the class labels.

## 3. Results

### 3.1. Correlation Analysis of Behavioral Data

Exercising the task every day, subjects continuously improved their performance, reflected in a monotonic decrease of the average tracking error shown in Figure 2A. The only exception was on day 7, when the introduction of ECG and EEG recording likely affected the experimental routine acquired during the previous days, leading to a transient decrease of task performance. Average performance was higher when subjects tracked individual targets compared to when they collaborated to track the target jointly. On days 7 and 8 the average tracking error was 49.8/55.6 pixels in the individual/collaborative condition, respectively (*p* = 2.4466e^−4^, paired two-sided *t*-test). The subjects rated their own performance slightly higher in the individual than in the collaborative condition (6.3269 vs. 6.1319, *p* = 0.0429).

Participants mainly used the upper half of the 9-point scale (values 5–9) for assessing task performances, with 6 and 7 being the most frequent responses (Figure 2B). The distributions of the responses was nearly normal (Lilliefors test, *p*-values for the four ratings between 0.01 and 0.021).

**Figure 3:**
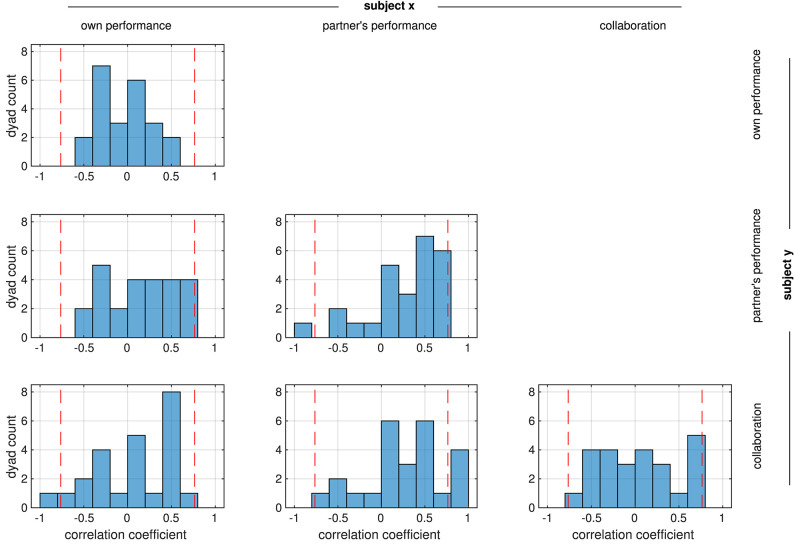
Distribution of correlation coefficients between the ratings from the two partners in each dyad. All possible correlations were calculated, e.g., the top panel shows the correlation coefficients between the ratings of own performance (R1) from the two partners. Red dashed lines mark the strength beyond which correlations are significant (*p* < 0.05, FDR-corrected).

We analyzed the relation between the performance assessments of the two partners in the collaborative condition by calculating Pearson correlation coefficients between all possible combinations of the ratings R1–3. For most rating combinations, correlations were more or less evenly distributed across the negative and positive ranges (Figure 3). Only correlations between mutual ratings of the partner's performance (R2 subject x vs. R2 subject y, center panel) and between ratings of the partner's performance and collaboration (R2 subject x vs. R3 subject y, middle panel in the bottom line) appeared to be significant (medians different from zero: *p* = 0.013 and *p* = 0.015, respectively, Wilcoxon signed rank test). Despite this prevalence of positive correlations in the mutual assessment of the partner's performance, the rather flat distribution of correlations between ratings of the collaboration of the partners (R3 subject × vs. R3 subject y, lower right panel) indicates that in most dyads, a feeling of ‘good collaboration’ was rarely reciprocated by the partner, and in some dyads this feeling even was inverse.

**Figure 4:**
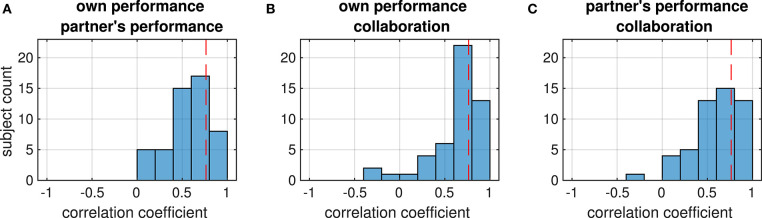
Distribution of correlation coefficients between the ratings (printed above each histogram) from each subject. Red dashed lines mark the strength beyond which correlations are significant (*p* < 0.05, FDR-corrected). The four panels show the distribution of correlations between **(A)** ratings of the own and the partner's performance on the own axis (collaborative condition), **(B)** the partner's performance rating and the tracking error on the partner's axis, **(C)** ratings of collaboration and the Euclidean tracking error, **(D)** own performance rating and tracking error on the own axis (individual condition).

Correlations were weak in general; only in a few dyads they reached a threshold of *p* < 0.05 (FDR-corrected) which is marked by the dashed lines in Figure 3. This suggests that subjects in a dyad rated the performance independently of the partner. We therefore investigated whether there was a systematic relation between the ratings within each subject instead. In contrast to the relation between the ratings in the dyad, most ratings from an individual subject were positively correlated (median different from zero: all *p* < 1e^−8^, Wilcoxon signed rank test), reaching a significance threshold of *p* < 0.05 (FDR-corrected) in several subjects (Figure 4).

**Figure 5:**
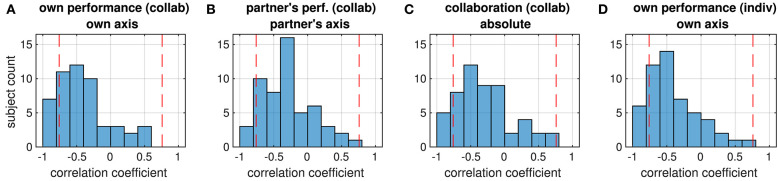
Distribution of correlation coefficients between ratings (given in the first line of the title) and objective performance (tracking error, second title line) of all subjects. Red dashed lines mark the strength beyond which correlations are significant (*p* < 0.05, FDR-corrected). Correlations are mostly negative because subjects were asked to rate higher performance, which corresponds to smaller tracking errors, by larger values. The four panels show the distribution of correlations between **(A)** own performance rating and tracking error on the own axis (collaborative condition), **(B)** the partner's performance rating and the tracking error on the partner's axis, **(C)** ratings of collaboration and the Euclidean tracking error, **(D)** own performance rating and tracking error on the own axis (individual condition).

A third set of correlation analyses was run to figure out in how far the subjects' ratings reflected actual task performance. From the trials in the collaborative condition, we calculated the correlation of the own performance ratings with the tracking error along the subject's axis, the partner's performance rating with the tracking error along the partner's axis, and the collaboration rating with the absolute tracking error. Whereas most subjects showed a negative correlation between the tracking error and their ratings (median different from zero: all *p* < 1e^−5^, Wilcoxon signed rank test), only in a few of them this correlation was significant, suggesting that most subjects were hardly objective in the assessments of their own performance, their partner's performance or the success of their collaboration (Figure 5). In addition, we could not observe any significant difference in the distribution of correlation coefficients between the four ratings (all *p*> 0.82, Wilcoxon signed rank test).

### 3.2. Analyzing the Relation Between Ratings and Individual Autonomic Parameters

**Figure 6:**
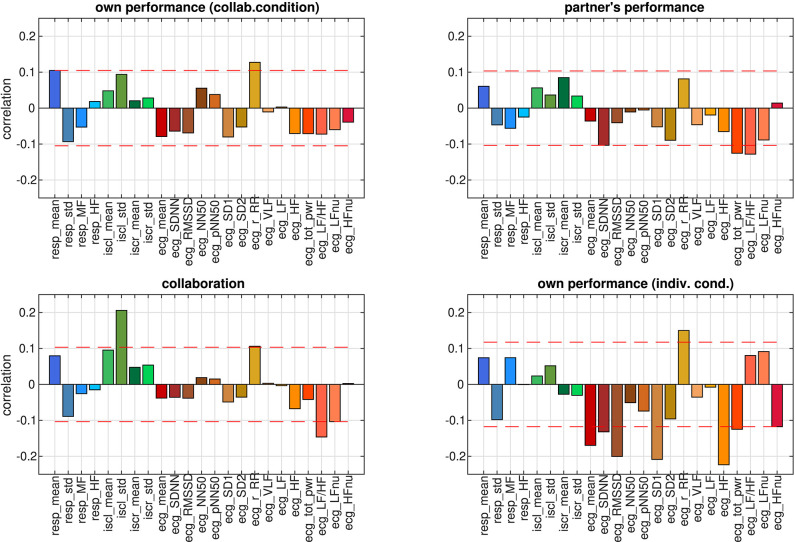
Correlation coefficients between individual autonomic parameters and ratings. RR parameters have blue hues, EDA parameters green colors, and HRV parameters shades of red. Dashed red lines visualize the strength beyond which correlations are significant (*p* < 0.05, FDR-corrected).

Following a conventional approach for studying the properties of autonomic parameters in cognitive tasks, we analyzed correlations between each of the 23 parameters that were used in this study (see Tables 1–3) and the ratings. From the set of HRV parameters, one ore more were significantly correlated with each of the four different ratings (see Figure 6). In particular the r_RR parameter (correlation coefficient in the Poincaré plot) correlated with ratings of own performance in the collaborative and individual conditions as well as with ratings of collaboration. In the individual condition, 8 of the 15 HRV parameters were correlated with ratings of own performance, whereas for the collaborative condition, only 3 or less parameters correlated with the ratings. Collaboration was the only rating that correlated with one of the EDA parameters, the standard deviation of the skin conductance level (iscl_std). Own performance in the collaborative condition was the only rating that correlated with one of the RR parameters, the mean respiration rate (resp_mean). About half of the parameters however did not show significant correlations with at least one of the four ratings.

### 3.3. Predicting Ratings From Autonomic Parameters and Objective Performance

**Figure 7:**
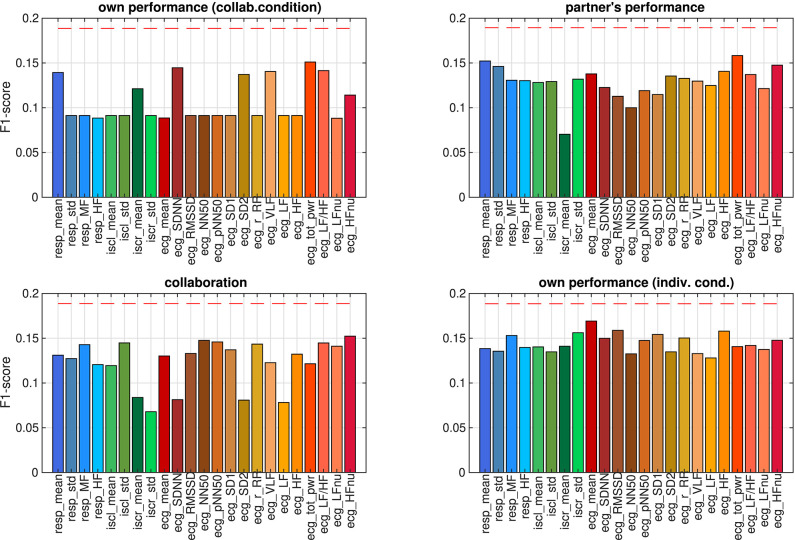
F1-scores of predicting ratings from individual autonomic parameters, estimated using leave-one-sample-out cross-validation. Dashed red lines mark the 0.05 quantile of obtaining the corresponding F1-score or higher by chance (FDR-corrected).

The correlation analysis in the previous section showed that some of the autonomic parameters bore a linear relation to at least one of the ratings. The correlations were calculated across all participants and trials. In the next step we explored to what extent this relation would enable a machine classifier to make predictions about single trials. We successively trained a classifier to predict ratings from each autonomic parameter individually and evaluated the performance by leave-one-sample-out cross-validation. Since samples in the data set were unevenly distributed across rating levels 5–9 (see Figure 2B), we could not employ accuracy for quantifying classification performance. For non-equally distributed target classes, classification accuracy may be a misleading quality measure, because a classifier could achieve high accuracy values by simply deciding for the most frequent class. Instead, we used F1-scores to compare the classification performances on different autonomic parameters. We determined the likelihood of observing these performance values when in fact there is no structure in the data by comparing them against the distribution of F1-scores on surrogate data. The result is shown in Figure 7. None of classification performances exceeded the chance level (all *p* > 0.05, FDR-corrected), that is, none of the ratings could be predicted from any of the autonomic parameters.

**Figure 8:**
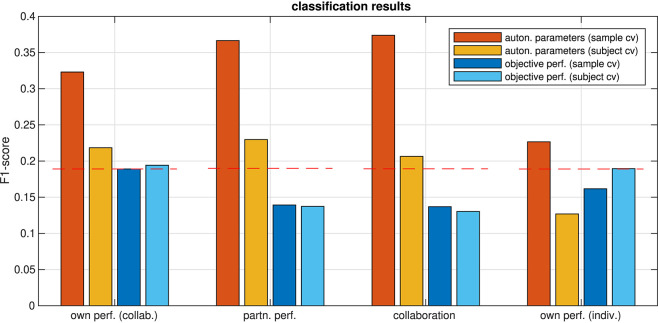
F1-scores of predicting ratings from aggregated autonomic parameters and from objective performance, estimated using cross-validation on a single sample (sample cv) and on all samples from one subject (subject cv). Dashed red lines mark the 0.05 quantile of obtaining the corresponding F1-score or higher by chance (FDR-corrected).

To investigate whether this situation could be changed when autonomic parameters are considered jointly rather than individually, we trained another set of models on feature vectors which were composed from subsets of the autonomic parameters. Starting from a feature set with all parameters, we eliminated one by one until classification performance did not improve further (backward feature selection). The classification results on the optimized parameter set are summarized in Figure 8. Prediction performance from the aggregated parameters was well above chance level for all ratings. Prediction of ratings was generally better in the collaborative than in the individual condition. To explore the stability of these findings, we also tested whether the models captured some general properties of the relation between ratings and the autonomic response across the population, which would allow it to make predictions about unseen subjects. We therefore ran a leave-one-*subject*-out cross-validation on the same parameter sets. Prediction performances were generally lower under this cross-validation method, which had to be expected since generalizing to a unseen subject is harder than generalizing to new samples when data from all subjects were already seen in the training. Nevertheless, this analysis revealed that the subjective performance evaluation of an unseen participant in the collaborative condition could still be predicted from the autonomic response of this participant above chance level, whereas this was not possible when the participants solved the task individually.

For the relation of the objective task performance measured by the tracking error to the subjective experience, the analysis in section 3.1 showed that correlations were significant only in a few participants (c.f. Figure 5), but that for most of them, the correlations were stronger than those for individual autonomic parameters (shown in Figure 6). This raised the question whether these stronger correlations could result in a better predictability of the subjective performance evaluation from the objective task performance than from individual autonomic parameters. The results of predicting ratings from the objective performance using both cross-validation methods are shown in Figure 8. Indeed, prediction performance of own performance ratings from the tracking error reached the chance level, which is a clear improvement compared to the prediction from individual autonomic parameters (c.f. Figure 7). However, when subjects collaborated, predicting ratings from objective performance was always inferior to the prediction from aggregated autonomic parameters. In contrast, the generalization capabilities of the model across subjects seemed to be better for the tracking error than for the autonomic response when subjects tracked targets individually. Numerical values of F1 scores and *p*-values are listed in Tables 4, 5.

**Table 4:** F1-scores, precision, and recall from leave-one-sample-out cross-validation and *p*-values from a randomization test.

**Rating**	**Classification from auton. param**.				**Classification from obj. perform**.			
	**F1-score**	**p**	**prec**.	**recall**	**F1-score**	**p**	**prec**.	**recall**
Own performance (R1, collab.)	0.32	0	0.43	0.26	0.19	0.048	0.18	0.19
Partner performance (R2)	0.37	0	0.47	0.3	0.14	0.866	0.11	0.18
Collaboration (R3)	0.37	0	0.46	0.31	0.14	0.905	0.11	0.18
Own performance (R1, indiv.)	0.23	0	0.23	0.23	0.16	0.461	0.14	0.2

**Table 5:** F1-scores, precision, and recall from leave-one-subject-out cross-validation and *p*-values from a randomization test.

**Rating**	**Classification from auton. param**.				**Classification from obj. perform**.			
	**F1-score**	**p**	**prec**.	**recall**	**F1-score**	**p**	**prec**.	**recall**
Own performance (R1, collab.)	0.22	0.001	0.22	0.22	0.19	0.027	0.19	0.2
Partner performance (R2)	0.23	0	0.25	0.22	0.14	0.889	0.11	0.18
Collaboration (R3)	0.21	0.008	0.18	0.24	0.13	0.96	0.11	0.17
Own performance (R1, indiv.)	0.13	0.984	0.14	0.11	0.19	0.045	0.18	0.2

### 3.4. Analyzing the Relevance of Individual Parameters

**Figure 9:**
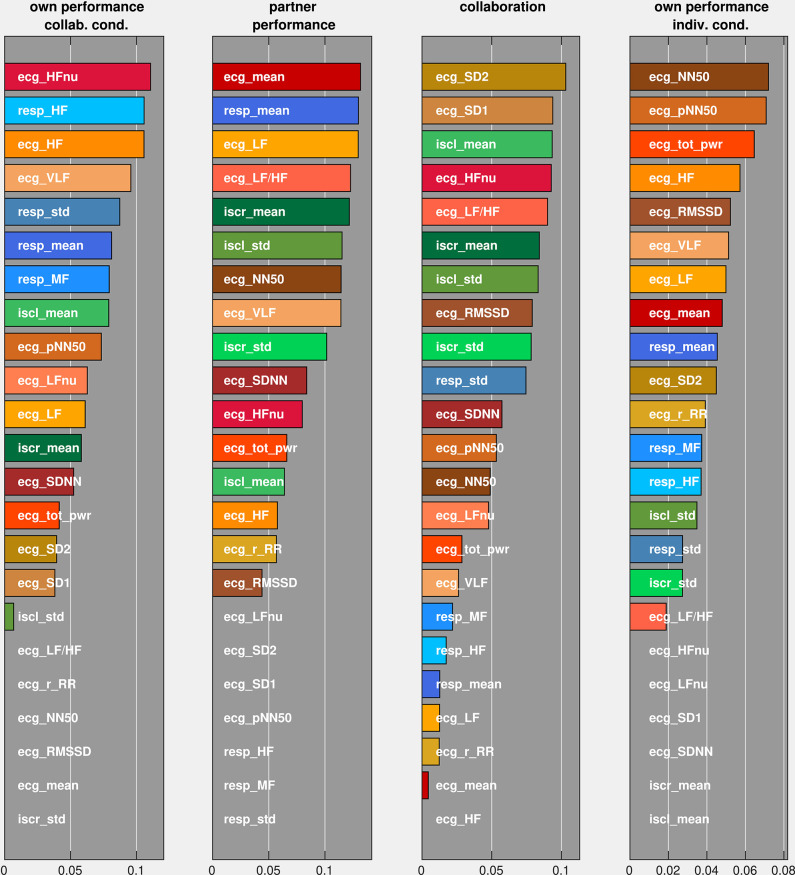
Average decrease of F1-scores (on the abscissa) under permutations of individual predictors (on the ordinate). Predictors were ordered according to their associated F1-score change.

Finally, we were interested in the importance of individual autonomic parameters in the optimized feature set for the prediction performance. We therefore permuted each parameter and ordered them according to the incurred decrease in the model's prediction performance (Figure 9). Comparing the three groups of autonomic parameters, we observed that all RR parameters are among the seven most important parameters for the prediction of own performance in the collaborative condition, whereas they rank lower in predicting the remaining ratings (with the exception of the mean breathing rate for predicting the partner's performance). EDA parameters play a role in predicting the partner's performance and collaboration but are less important in predicting own performance. For all ratings, different combinations of HRV parameters have the strongest influence on the prediction performance. From the set of HRV parameters, frequency-related parameters (HF, HFnu, LF, LF/HF, VLF) seem to be critically involved in the prediction of own and partner performance in the collaborative condition, whereas prediction of collaboration and own performance in the individual condition relies more on parameters which capture the regularity of the heart beat intervals in the time domain (NN50, pNN50, SD1, SD2).

## 4. Discussion

This study investigated the relation between the subjective assessment of performance and activity of the autonomic nervous system indexed by HRV, RR, and EDA in a joint target-tracking task. Ratings of collaboration, partner and own performance, which were reported by the subjects after each trial, were highly correlated within each subject. This indicates that subjects did not differentiate much between the individual contributions inquired by the different questions and possibly rated them on the basis of a general impression of the success in tracking the target instead. Partners within a dyad however rarely agreed upon the success of their collaboration or the performance of their partner. Hence, it seems that the experience of joint task performance was not shared among the partners. Whereas the joint target-tracking task and the instructions engaged many of the coordinating mechanisms that constitute joint action (Vesper et al., 2017) (e.g., monitoring, joint attention and shared gaze, haptic coupling, emotion understanding, and expression), critical components for making the task completion a joint experience may have been missing. The independent control of orthogonal axes was likely to impact the joint action goal as well as the task co-representation, leading to a collaboration experience that was not systematically reciprocated. Yet subjects reacted differently to the collaborative and to the individual condition, which became evident in the lower tracking error and higher ratings of their own performance in the individual condition. The current results should therefore be interpreted on the background of an experimental manipulation of joint attention (Maye et al., 2017) rather than joint action.

We compared the capability of predicting ratings from aggregated autonomic parameters and from the tracking error as an objective measure of task performance. We found that predictions from autonomic parameters generally were more reliable, in particular for ratings of the partner's performance and the success of the collaboration. Autonomic parameters were also more effective in predicting ratings of own performance, but the prediction performance was lower for trials in which the target was tracked individually than for joint tracking. The prediction performance for the own performance from the tracking error seemed to be less affected by the condition though. Taken together, our results suggests that the autonomic response is more informative for inferring the subjective experience in the collaborative condition and less so when interaction with the partner is not required. For the efficiency of the objective task performance, however, the difference is between assessments of own performance, no matter whether in a collaborative or individual context, and the evaluation of the contribution of the partner to achieving a common goal. These findings may provide support for efforts to increase social coherence by using realtime-feedback for enabling group members to co-regulate HRV coherency (McCraty, 2017).

With respect to the two hypotheses about the origin of the subjective performance assessment, our results suggest that H1 can not sufficiently well explain how subjects arrived at their ratings. H2 entails that the subjects' ratings were guided by a feeling about the performance, and that this feeling is modulated by visceral information. In particular in a collaborative setting, the autonomic response may be a good indicator for the outcome of this subjective performance assessment. The stronger coupling between ratings and these parameters in the collaborative condition suggests that subjects rely more on the internal state of their body when assessing the outcome in a collaborative task.

A crucial point of this study is that the relation between the autonomic response and the outcome of a subjective assessment could only be observed when the parameters were considered in an integrated manner. Traditional studies investigate the relation between a single (or a few) parameter(s) and the experimental paradigm. Quite often, these studies discriminate between fluctuations on a short time scale which are reflected in short-term components of the HRV (e.g., RMSSD, NN50count, SD1, or HF) and variability on a longer time scale as indicated by long-term components (e.g., SDNN, SD2, or LF), and interpret effects as shifts of the sympatho-vagal balance in the modulation of cardiac activity. This approach is followed on the background that long-term components, such as LF, and short-term components, such as HF, reflect primarily sympathetic and vagal modulations, respectively. Here, this approach did not reveal such an obvious systematic relation between any of the typical HRV parameters (Camm et al., 1996; Brennan et al., 2001) and ratings. However, considering the same parameters as elements of a feature vector and training a simple classifier allowed us to obtain a population model which predicted the result of the subjective assessment better than that of a model based on the objective performance. This indicates that there is a systematic relation between autonomic parameters and subjective performance evaluation that is shared across the population. Since we observed this relation only for aggregated parameters and not for individual ones, we cannot interpret the result with respect to only one of the mental-cognitive phenomena that are typically considered in the literature in relation to autonomic parameters. Yet, one should consider that sympathetic and parasympathetic activity are not always entirely antagonistic, rendering a simple concept of sympathetic-parasympathetic balance inadequate in complex cognitive, emotional, and behavioral situations.

Whereas, the results of our study show some potential for a better understanding of the embodied nature of subjective experience, the ramifications of the approach have to be elucidated in future investigations. An important issue in this respect is the demography of the cohort and the composition of the dyads. Participants in our study were young students, and a clear majority of them were females. As gender is known to affect the dynamics of autonomic parameters, it would certainly be interesting to find out whether and how this might impact the prediction capabilities of the proposed method. Other factors in this context which require systematic investigation are whether the partners in a team are from the same or different sex as well as their relationship. Another avenue for future research is probably the question in how far the findings in our study can be generalized across different tasks. Methodological difficulties notwithstanding, we think that the evidence for the importance of bodily signals in the emergence of subjective experience that we found in our study suggests that tasks which require more physical play than just flicking a finger may result in better prediction capabilities.

## 5. Conclusion

Existing studies have suggested a variety of interactions between cognitive and perceptual processes and individual autonomic parameters, but almost all of them concluded that the true relations are likely more complex. The machine-learning-inspired approach we suggest here may pave the way to understand such complex relationships. Our study suggests that the physiological activity indexed by autonomic parameters bears a relation to the subjective performance evaluation that can be stronger than that of the actual performance. This underlines the importance of considering bodily processes for understanding the mechanisms of social cognition.

## Data Availability Statement

The datasets generated for this study are available on request to the corresponding author.

## Ethics Statement

The studies involving human participants were reviewed and approved by the ethics committee of the medical association of the city of Hamburg. The patients/participants provided their written informed consent to participate in this study.

## Author Contributions

The experiments were conceived and designed by AE, MS, and AM. The experiments were performed by MS and AM. The data were analyzed by AM and JL. The paper was written by AM, JL, and AE. All authors contributed to the article and approved the submitted version.

## Conflict of Interest

The authors declare that the research was conducted in the absence of any commercial or financial relationships that could be construed as a potential conflict of interest.
